# Neuromorphic computation with a single magnetic domain wall

**DOI:** 10.1038/s41598-021-94975-y

**Published:** 2021-08-02

**Authors:** Razvan V. Ababei, Matthew O. A. Ellis, Ian T. Vidamour, Dhilan S. Devadasan, Dan A. Allwood, Eleni Vasilaki, Thomas J. Hayward

**Affiliations:** 1 Department of Material Science and Engineering, University of Sheffield, Sheffield, S1 3JD UK; 2 Department of Computer Science, University of Sheffield, Sheffield, S1 4DP UK

**Keywords:** Magnetic properties and materials, Magnetic devices, Computational science

## Abstract

Machine learning techniques are commonly used to model complex relationships but implementations on digital hardware are relatively inefficient due to poor matching between conventional computer architectures and the structures of the algorithms they are required to simulate. Neuromorphic devices, and in particular reservoir computing architectures, utilize the inherent properties of physical systems to implement machine learning algorithms and so have the potential to be much more efficient. In this work, we demonstrate that the dynamics of individual domain walls in magnetic nanowires are suitable for implementing the reservoir computing paradigm in hardware. We modelled the dynamics of a domain wall placed between two anti-notches in a nickel nanowire using both a 1D collective coordinates model and micromagnetic simulations. When driven by an oscillating magnetic field, the domain exhibits non-linear dynamics within the potential well created by the anti-notches that are analogous to those of the Duffing oscillator. We exploit the domain wall dynamics for reservoir computing by modulating the amplitude of the applied magnetic field to inject time-multiplexed input signals into the reservoir, and show how this allows us to perform machine learning tasks including: the classification of (1) sine and square waves; (2) spoken digits; and (3) non-temporal 2D toy data and hand written digits. Our work lays the foundation for the creation of nanoscale neuromorphic devices in which individual magnetic domain walls are used to perform complex data analysis tasks.

## Introduction

Classifying or predicting complex time-dependent signals (e.g. speech, financial data, or weather patterns) are challenging computational tasks^1^. Recurrent neural networks (RNNs), where the network connectivity contains loop-like structures, are a powerful method for solving such tasks but the inherent temporal dependencies can make them expensive to train and optimize. As an alternative, reservoir computing (RC) is a neuromorphic computing paradigm that circumvents these issues by using a RNN with fixed synaptic weights (known as the reservoir), typically implemented algorithmically in software, connected to a single, easily trainable readout layer^2,3^. However, more energy efficient implementations of RC are possible if the software RNN reservoir is substituted with a physical system with the correct properties, namely a non-linear response to input signals and a fading memory of previous inputs^4,5^.

Nanoscale magnetic systems are excellent candidates for use as physical reservoirs. Their dynamical complexity means that they commonly exhibit highly non-linear responses to input, while their non-volatility provides memory of previous inputs. Furthermore, their use in both magnetic hard disk drives and magnetic random access memories have provided well-established routes to data input and output, and integration with existing CMOS technology^6^. Together these properties have inspired numerous proposals for both hardware reservoirs^7–14^, and a broader range of neuromorphic devices based on nanomagnetic technology^11,15–17^. In particular, magnetic tunnel junction-based spin torque oscillators have previously been used for a range of neuromorphic applications, mostly notably as a dynamical reservoir^7,18^ and for vowel recognition^19^, due to their highly non-linear responses to inputs with an inherent dynamical memory^20^.

Domain walls in magnetic nanowires have long been considered for use as data carriers in both “racetrack memory” memory devices^6,21^ and logic networks^22–24^. Many proposed approaches to these have been inhibited by the complex magnetization dynamics of domain walls, which ultimately lead to unreliable device operation^25,26^. Other studies have proposed non-volatile, DW-based neurons and synapses that could be integrated into CMOS devices to create hybrid neuromorphic computing platforms^27–29^. Applications in these areas may be more robust against stochasticity than those in conventional memory and logic, due to the intrinsic error tolerance of neuromorphic approaches to computation. However, we propose that the complex, oscillatory dynamics of DWs may be directly exploited as a functional feature when used in the framework of RC, where non-linear responses to input are essential.

In this paper, we use micromagnetics and a 1D collective coordinates model to demonstrate that individual domain walls trapped between artificial defect sites in planar magnet nanowires can exhibit dynamics that are suitable for use as hardware reservoirs. We first demonstrate how complex non-linear dynamics of a domain wall in our chosen structure vary with applied magnetic field. We then go on to illustrate how optimized dynamics can be used for RC to tackle three machine learning problems: (1) the classification of sine and square waves; (2) the classification of spoken digits and (3) the classification of binary 2D data and hand written digits. Finally, we discuss the challenges that will need to be overcome to realize RC devices based on DWs, and the advantageous properties that such devices would possess.

## Results

**Figure 1:**
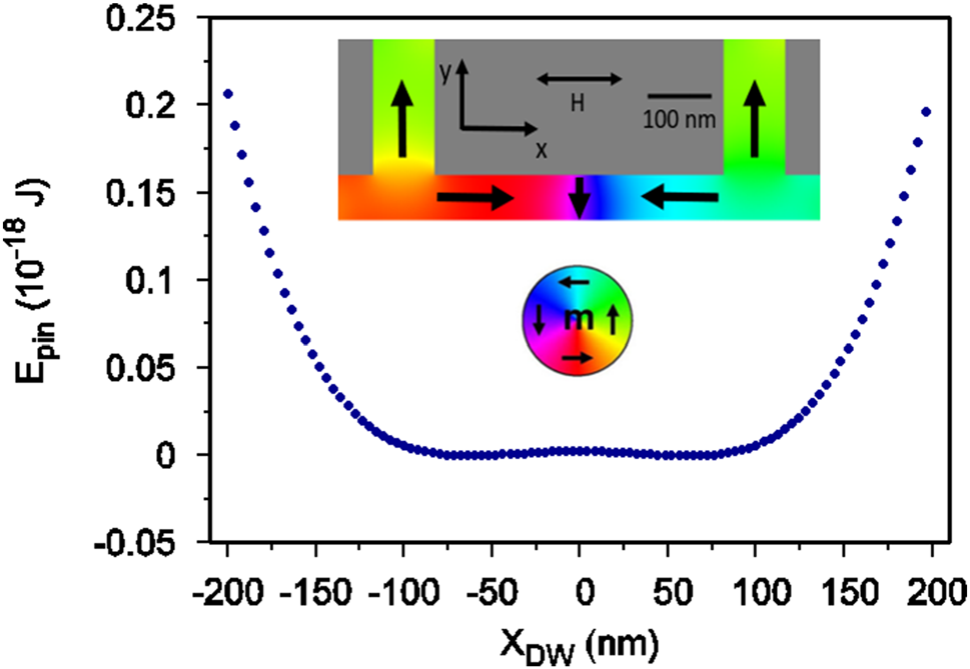
The pinning energy profile, *E*(*X*), for a transverse domain wall between two anti-notches extracted by fitting the micromagnetic simulations according to reference^30,31^. The inset shows a schematic representation of the single domain wall in a magnetic nanowire with two anti-notches placed symmetrically at 350 nm distance edge-to-edge.

As previously demonstrated by Pivano and Dolocan^30^, the dynamics of DWs when trapped between two artificial pinning sites can be highly non-linear for certain amplitudes and frequencies of the driving field. These properties make it a possible candidate for reservoir computing despite it’s relative simplicity. In this work a nanowire with two symmetric anti-notches was considered. The geometry of the modelled DW oscillator is illustrated in the inset of Fig. 1 where the colour contrast illustrates the symmetric, transverse magnetization configuration the DW formed. It consisted of a (600 nm × 50 nm × 5 nm) nickel nanostrip with two (70 nm × 150 nm) anti-notches with edge-to-edge spacing of 350 nm placed about its center. A transverse DW was initialized in the centre of the nanowire such that its magnetization opposed that of the anti-notches.

The effect of this structure was to pin the DW in an effective potential well where the DW could not escape past the anti-notches. Figure  1 shows the pinning potential calculated using micromagnetics with the anti-notches 350 nm apart. The pinning potential varied with the position of the DW along the length of the nanowire, *X*, and exhibited a clear double well shape that could be simplified as $E_\text {pin}(X) = aX^2 + bX^4$. This double well shape arose from the increase in exchange energy as the DW moved closer to the anti-notches, reflected in the confining $X^4$ potential, while the complex interaction of exchange and demagnetizing energy gave rise to a barrier in the centre of the structure that was modelled by the $X^2$ term.

In the following, we will demonstrate how the DW oscillator could be used as a hardware-based reservoir. We will first present an exploration of the range of the dynamics exhibited by the system, and show that the results of the collective coordinates model agree well with those of more physically detailed micromagnetic simulations. Following this we show how utilizing optimized DW dynamics with a time-multiplexed reservoir computing method allows the DW oscillator to perform a range of classification tasks.

### Domain wall oscillator dynamics

The dynamics of the DW were driven by sinusoidally-varying magnetic fields applied along the nanowires length. The DW dynamics of the system described above have been previously modelled by Pivano and Dolocan^30^, who explored the range of dynamics exhibited by the DW using both micromagnetic simulations and a 1D collective coordinates model. Here, we primarily adopt their latter model for computational efficiency. In the 1D model the DW was represented by its position along the wire, *X*, and tilt angle of the DW centre, $\psi$. The full details of the 1D collective coordinates model is given in the Methods section.

**Figure 2:**
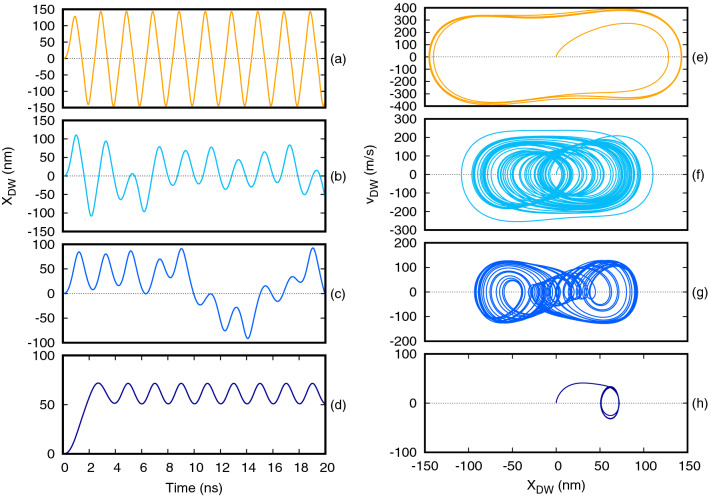
Simulated dynamics of the DW using 1D model for four different amplitudes of the applied magnetic field. (**a**)–(**d**) Show the time evolution of the DW’s position at (**a**) 1500 A/m, (**b**) 1000 A/m, (**c**) 500 A/m, (**d**) 100 A/m. The graphs (**e**,**f**) show the corresponding phase-space diagram for each value of the amplitude at a fixed frequency of the oscillating field of $f={500}\,\text {MHz}$.

Figure 2 illustrates typical DW trajectories when the DW oscillator was driven by an oscillating fields of amplitude (a) $H={1500}\,\text {A/m}$, (b) 1000 A/m, (c) 500 A/m, and (d) ${100}\,\text {A/m}$. We impose initial conditions of: $\psi (t=0)=0$ and , $X(t=0)=0$. The frequency of the oscillating field was $f={500}\,\text {MHz}$. As the applied field was decreased the the dynamics changed from non-linear harmonic motion at high fields (Fig. 2a), to chaotic motion at intermediate fields (Fig. 2b,c), before returning to harmonic motion at the lowest fields (Fig. 2d). The harmonic dynamics at the highest and lowest fields were differentiated by the localisation of the DW within the bistable potential well presented by the anti-notches: for the lowest fields the DW was trapped in a single well, while for the highest fields it traversed both sides of the energy landscape.

Figure 2e–h present phase-space diagrams corresponding to the time-domain data shown in Fig. 2a–d. As expected, in the harmonic regimes trajectories rapidly converged to well-defined, periodic orbits in phase space, thus outlining the attractor of the system for given applied field amplitude (Fig. 2e,h). In contrast to this, in the chaotic regime the trajectories were aperiodic and did not converge to well-defined orbits, instead they traced out strange attractors, which are characteristic features of chaotic systems (Fig. 2f,g). The dynamics observed here were phenomenological similar to those of a Duffing oscillator, and are in agreement with the results previously presented by Pivano et al.^30^.

**Figure 3:**
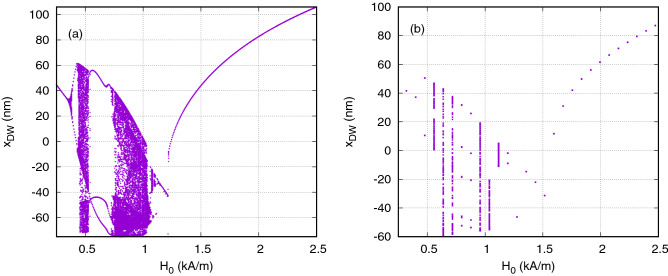
Bifurcation diagrams for Ni at 500 MHz using 1D model shown in graph (**a**) and micromagnetic simulations shown in graph (**b**).

In order to more thoroughly characterize the various dynamical regimes of DW oscillator as a function of the applied field amplitude we constructed a bifurcation diagram. We achieved this by sampling the position of the DW once per applied field period over a total of 200 field cycles. In these diagrams the regimes of motion could be deduced from the number of distinct data values that were visible for a given field value. Where a single data value was visible, this indicated harmonic motion, as the system returned to the same position once per field cycle. Where a finite number of data values were visible, this corresponded to multi-period motion, where the period of the DWs’ motion was an integer multiple of that of the applied field. Finally, where bands of data values were visible, this corresponded to chaotic motion, with the DWs motion being aperiodic, but constrained to within well defined limits. Figure 3 presents bifurcation diagrams obtained using (a) the collective coordinates model and (b) micromagnetic simulations. In the collective coordimates model chaotic dynamics occurred in a field range $H_0 \approx {0.4}\,\text {kA/m}$ to $\approx {1.05}\,\text {kA/m}$ with clear period-doubling approaches to chaos occurring at its periphery. A period three window was observed in middle of the chaotic region. Below 0.35 kA/m the oscillations were harmonic within one side of the bistable potential, while for fields >1.25 kA/m harmonic motion occurred across the full potential landscape.

The bifurcation diagrams from the two simulation techniques were in good agreement with each other, showing the same basic distribution of dynamical regimes. There was reasonable quantitative agreement between the fields at which transitions between regimes of dynamics occurred, although these appeared to occur at slightly higher fields in the micromagnetic model, perhaps suggesting slight inaccuracies in our description of the pinning potential in the collective coordinates model. The overall similarity between the approaches, however, reinforced the validity of using the collective coordinates model to further explore both the dynamics of the system and its suitability for use as a reservoir. The bifurcation diagrams clearly demonstrated the richness of the DW oscillator dynamics and provided a reference for understanding how the magnitude of the applied field could be tuned to bring the oscillator into different dynamical regimes.

### Reservoir computing with DW oscillator

Having explored the inherent dynamics of the system, we moved on to investigating the performance of the pinned DW oscillator as a reservoir. In the reservoir computing approach, the complexity of training a recurrent network is avoided by considering a fixed reservoir that transforms the input in such as way that the output is linearly separable. Thus, training is only needed on this output, which can be done with a simple linear method (perceptron). The assumption is that the reservoir dynamics are rich and can easily map the input into a higher-dimensional representation separable by a hyperplane. Typical approaches employ simulated neural networks with fixed random connectivity, known as echo state networks^2^, and therefore have a wide input/output dimensionality which is not possible with a single DW oscillator.

**Figure 4:**
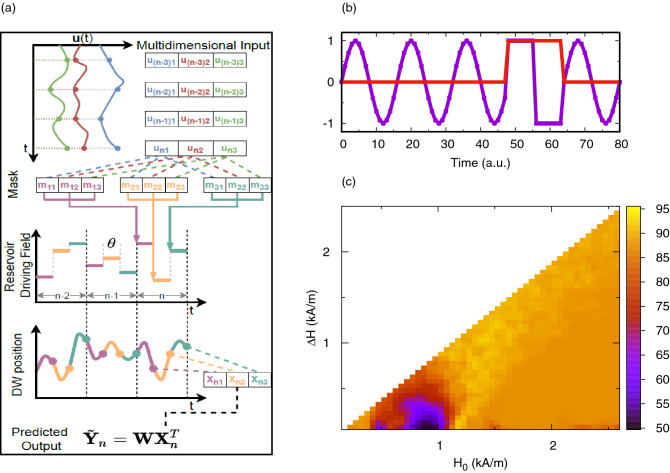
Schematic representation of the reservoir computing process used illustrated by figure (**a**). Initially a multidimensional signal ${\mathbf {u}}(t)$ is sampled to give discrete values which is stored as $N_s$ by $N_\text {in}$ array. Each sample vector is multiplied by a random mask matrix to project the input dimensions randomly as the driving magnetic field for each virtual node. The field for each node in turn is applied to the DW oscillator for a duration of $\theta$ and when all virtual nodes for a single sample have been applied, the next sample is used. The DW position is sampled at the end of each virtual node time segment to form a matrix of transformed values ${\mathbf {X}}$ to which the predicted output $\tilde{{\mathbf {Y}}}$ is calculated using the output weights. Effect of $H_0$ and $\Delta H$ on the classification of sine and square waves. (**b**) Shows a sample input (blue) and desired out (red). Discretized data points as part of the sine wave have a desired output of 0 while the square wave has + 1. (**c**) Illustrates the accuracy of the sine and square classification task as a function of the base driving field amplitude, $H_0$, and stimulus amplitude, $\Delta H$, for $N_v=8$.

A method to construct a complex reservoir with only a single dynamical object was presented by Appeltant et al.^32^ and later used by Torrejon et al.^7^ to perform reservoir computing with a single magnetic tunnel junction. In this method rather than having multiple physical neurons to input and read out from, the system’s states at different time intervals are used as ‘virtual’ neurons i.e. input/output is multiplexed in time. In this way the virtual neurons are connected sequentially in time and each input dimension is randomly combined with an input mask to drive the reservoir dynamics at each virtual neuron. This temporal connection is equivalent to a directed ring structure network. The full details of are given in the methods section and a summary is shown schematically in Fig. 4a. As shown in the schematic, the multidimensional input $u_{nk}$ is multiplied by the random mask $m_{jk}$ to give a total input for each of the $N_v$ virtual neurons. This is then scaled into a driving magnetic field amplitude $H_{jk}$ using two parameters; the base amplitude, $H_0$, and input scaling, $\Delta H$. This field is then applied to the reservoir as an oscillating field for a duration $\theta$ for each virtual neuron. Sampling the DW position, or RMS position depending on the task, at the end of each time segment gives a transformed value ${\mathbf {X}}_n^T$ of the input for which a linear perceptron is used to give a predicted output value as ${\tilde{\mathbf {Y}}}_n = {\mathbf {WX}}_n^T$. Ridge regression (Eq. 11) is then used to fit the weights to the desired output by minimising the mean squared error with L$_2$ regularization that is controlled by the parameter $\lambda$, which was optimized by training the output for multiple values of $\lambda$ and selecting the value which gave the lowest error on the test data set (not an additional validation set).

#### Sine-square classification

The first task we considered was a simple time-domain task: the classification of sine and square waves^18^. The input was a random series of either full period sine or square waves discretized into 16 sample points per period. The aim of the task was to classify each data point with a desired output of 0 for the samples that were part of the sine wave or 1 for parts of the square wave. An example input and desired output sequences are shown in Fig. 4b. A sequence of 80 waveforms (1280 datapoints) in total was used, which was split evenly into a training and a test sets (640 data points in each). The number of virtual neurons, $N_v$, and the duration of these neurons, $\theta$, was tuned to optimize the connectivity of inputs through the fading memory of the reservoir. The mask took values − 1, + 1 and by definition the input values were within the range − 1 to 1, so the transformed input operated in the field range $H_0 - \Delta H$ to $H_0 + \Delta H$. Since there was only 1 input value at each time step the mask provided a perturbation to the sequence so as to trigger different dynamics^33^.

While this was a simple task, it required non-linearity and memory from the reservoir since a linear perception alone cannot separate the + 1 and − 1 data points from each class. To give the reader a baseline for the performance, randomly selecting the output would have achieved an accuracy of 50%, while a linear output layer without the reservoir that classified the inputs based on whether they were higher or lower than a set threshold would have reached a theoretical maximum performance of 68.75% which corresponded to setting the threshold just below + 1 and thus correctly classifying 7/8 of the sine wave and 4/8 of the square wave data points.

As discussed previously, the amplitude of the magnetic field could be used to tune the dynamics of the system into any of the possible harmonic, chaotic and multi-periodic regimes of motion. In the following, we present simulations that demonstrate the importance of these regimes of motion on the classification accuracy. We varied both the base amplitude of the driving field, $H_0$, and the scaling of the input signal, $\Delta H$. The reservoir performance, measured as $\%$ classification accuracy, over a range of $H_0$ and $\Delta H$ is illustrated in Fig. 4c for a fixed driving field frequency of 500 MHz. Initially, we selected $N_v=8$ virtual neurons, each with a duration $\theta ={0.5}\,\text {ns}$, giving a total time of $\tau = {8}\,\text {ns}$ per input sample, while testing the field parameters. Here, classification accuracy is reported from the test data set of 40 waveforms (640 data points) that was separate to those used during training. The values reported in the figure correspond to an average classification accuracy for ten different, random input masks.

Due to the different dynamics observed at different fields, the performance of the reservoir varied significantly over the field ranges studied. A region of high performance was observed at around $H_0 = {1.25}\,\text {kA/m}$ where an average accuracy of $(95 \pm 5)$% was achieved. Importantly, we observed that, in this region, certain realizations of the mask did reach a classification accuracy of 100%, while most were slightly lower. Since the mask was a random matrix, the cases where 100% accuracy was reached could be considered optimal masks, although we were unable to discern any empirical rules for generating an optimal mask. Including a larger number of virtual neurons increased the probability of including optimal features in the mask.

In contrast to this, classification accuracy was poor when field amplitudes were confined to those producing chaotic dynamics. A huge drop in classification accuracy was observed for $H_0 \approx$ 0.75 to 1 kA/m and for $\Delta H$ < 0.5 kA/m. This correlated with the chaotic regime of dynamics shown in Fig. 3a, indicating that the loss of classification accuracy was likely due to the high degree of non-linearity in the dynamics restricting the ability of the reservoir to generalize input signals.

It is interesting to note that the region of best performance lay at the boundary of the chaotic region of poor performance (around $H_0 ={1.2}\,\text {kA/m}$ in Fig. 4 and so the reservoir operates best in a region on the “edge of chaos” as has previously been suggested would be expected to be the case^32^. From the bifurcation diagram, shown in Fig. 3, we can see that beyond the chaotic regime there was initially a highly non-linear dependence of the sampled domain wall position on the applied field amplitude, which we would expect to help the reservoir separate different classes of input. Beyond this region, the non-linearity of the oscillator’s response gradually decreases (as evidence by the decreased rate of change of the gradient of the bifurcation diagram with H). Hence, larger values of $\Delta H$ were required to obtain the best classification accuracy: with $\Delta H$ small and $H_0$ large the response would appear linear, but when $\Delta H$ spanned a large field range a reasonable degree of non-linearity could be obtained.

**Figure 5:**
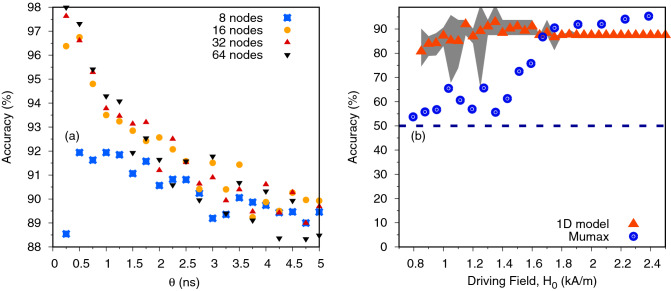
Graph (**a**) shows the dependence of the test set classification accuracy on $\theta$ for different number of virtual neurons where $\Delta H$ is kept at 750 A/m and $H_0$ is 1700 A/m. Graph (**b**) shows the test set classification accuracy of the sine and square task using 1D model and mumax$^3$ as a function of $H_0$. The input scaling was $\Delta H$ = 557 A/m. The results for the 1D model are averaged over 7 different random masks and the shaded grey area shows the minimum to maximum range of the accuracy. The dashed line shows the threshold of 50% accuracy which can be obtained with a random classifier.

Figure  5a shows how the accuracy varied with the number of virtual neurons and neuron duration ($\theta$). Since the driving field frequency was 500 MHz, a neuron duration of 2 ns corresponds to 1 neuron per cycle. In general a shorter neuron duration and a higher number of virtual neurons led to a higher classification accuracy, except for 8 virtual neurons where $\theta = {0.25}\,\text {ns}$ showed a decrease in performance. Higher accuracy could be achieved for a higher number of neurons due to the increased number of trainable parameters and the increased probability of generating ‘optimal’ sequences in the random mask. Increasing $\theta$ reduced connectivity between subsequent virtual neurons due to the system’s fading memory and thus reduced performance in all cases. Since the full time between input samples increased as $\tau = N_v \theta$, so that when both a long $\theta$ and high number of neurons was considered, the relative time between successive inputs was longer. When this time was long the interaction between successive inputs was low and may have reduced performance due to the weaker mixing of the inputs. This would explain the why the performance with 64 neurons in Fig. 5a dropped below the other sizes despite the increased number of weights.

Next we compared the results using the collective coordinates model to those obtained using the more complex micromagnetic model to validate our findings on a more realistic case. Due to the computational cost of simulating the micromagnetic system, we restricted the number of virtual neurons to eight with $\theta ={0.5}\,\text {ns}$ for these simulations. The micromagnetic reservoir computing was performed in the same manner as described in the previous section using mumax$^3$. Figure 5b compares the classification accuracy obtained from the 1D model against that obtained from equivalent micromagnetic simulations. The classification accuracy is presented as a function of $H_0$ with fixed $\Delta H = {557}\,\text {A/m}$. For the 1D model, the accuracy was averaged over 7 random masks and the shaded area on the figure shows the range of minimum to maximum accuracy. Both models converged to a classification accuracy > 85$\%$ at higher values of $H_0$, and exhibited a reduction in classification accuracy at low values of $\Delta H$, although the latter was more pronounced in the micromagnetic model. We believe that the reason for these differences can be observed in Fig. 3a, which compares the bifurcation diagrams of the 1D and micromagnetic models. The transitions between regimes of dynamics occurred at slightly higher fields in the micromagnetic model than in the 1D model. This meant that for the micromagnetic model our simulations explored a wider range of fields within or, close to the boundary of, chaotic dynamics where classification accuracy was apparently poorer. The micromagnetic model also exhibited a more substantial reduction in classification accuracy in these regimes which may have been the result of the more complex (and physically realistic) nature of the micromagnetic model. The shaded area shows the minimum to maximum range of the accuracy when different masks were used for the 1D model showing that the mask can reduce the accuracy in the chaotic regime. Since a single realization of the mask was used for the micromagnetic calculations, the accuracy could be improved with a different mask but was still consistently outside the range of the 1D model indicating that details present in the micromagnetic model, such as different demagnetizing and exchange energy or changing domain wall profile, were causing a reduction in the accuracy.

#### Spoken digits recognition

Having demonstrated the feasibility of performing a simple classification task with the DW oscillator system, we explored its capabilities to tackle a real world task: the classification of spoken digits. For this task, four speakers from the Free Spoken Digits Dataset (FSDD)^34^ were used, each speaker contained 50 utterances of each digit 0 to 9 thus totalling 2000 samples. Half of the data set was used for training and the other half as an independent test set. Each utterance was recorded at 8 kHz and the input pre-processed by transforming the audio waveform into a spectrogram, thus obtaining 64 frequency bands across 16 timesteps. Thus, the input for a single utterance was comprised of 1024 data points whose magnitudes were in the range $u_{nk} = [0,1]$. We adopted a concatenation approach where all 16 timesteps for one frequency band were fed into the reservoir before moving to the next frequency band. No input mask was used (i.e only one virtual neuron was used) since there were already a large number of outputs available to the classifier and additional virtual neurons in this context separated each input timestep by a time $N_v \theta$ on the reservoir but in this case it was important to allow the input at successive timesteps to interact through the fading memory. Our approach here replicated the preprocessing and data input approach used successfully in a previous publication^13^.

Input data were again fed into the reservoir according to Eq. (7). The applied field frequency was set at ${500}\,\text {MHz}$ and the magnitude of the driving field, $H_0$, was set to ${1000}\,\text {A/m}$. Each input value was held on the the neuron for $\theta = {0.5}\,\text {ns}$, as this was found to give good performance for the previous task. The DW’s position was reset to $(X(t=0)=0)$ after every utterance, since only memory within the utterance was important for classifying the digits.

To generate output data, ${\mathbf {X}}_n$, from the reservoir the RMS DW position was averaged over the neuron duration, which represented the amplitude response of the oscillator. When the whole utterance had been transformed, it was then sampled to give a set of reservoir states that were then concatenated into a single output vector. For all 64 frequency bands *M* timesteps were evenly sampled from the reservoir output giving $N_W = 64M$ possible output weights. An additional bias weight was intrinsically included in the Ridge regression fitting routine. In this way we could vary the number of output weights to allow the readout to access more or less temporal information. For classification, the desired output for each input sample were represented as a vector of size of $N_\text {out}=10$ for which all the elements were “0” apart from the element’s whose index that matched the input class which was “1”. The predicted output had the same shape and the classified digit label was taken as the index of the output element with the largest value.

**Figure 6:**
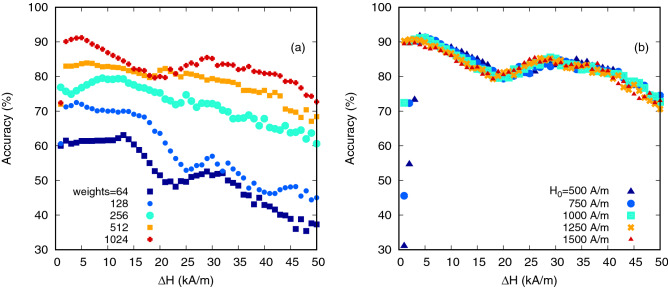
The dependence of the spoken digits recognition rate as a function of the amplitude of the input signal, $\Delta H$. Graph (**a**) shows the dependence at different number of weights where the amplitude of the driving field, $H_0$, is kept constant at ${1000}\,\text {A/m}$ and graph (**b**) show the same dependence for different values of $H_0$ at 1024 weights.

Figure 6 presents the performance of the DW oscillator for the spoken digit recognition task. Data are shown for 64, 128, 256, 512 and 1024 output weights, with higher numbers of weights representing higher-resolution sub-sampling of the reservoir output. The best performance was obtained for the maximum number of weights, i.e. where the dimension of ${\mathbf {X}}_n$ was equal to the input samples provided to the reservoir. Increased numbers of weights provided more temporal information to the output perceptrons, i.e when 1024 weights were used the whole of the input spectrogram had been collected after being transformed by the reservoir. Under these optimal conditions the classification accuracy was in excess of 90%, compared to 83% when the input data was passed directly to the linear readout layer (i.e the reservoir was bypassed). These results were broadly comparable to recent demonstrations of speech recognition with spin-torque oscillators, where an accuracy of 65.2% was obtained with spectrogram pre-processing, albeit on a different dataset (TI-46)^35^. With a lower number of weights the task was more challenging since the reservoir had to rely on its own fading memory to provide a temporal ‘mixing’ of the inputs to improve the information supplied to the readout layer. Since we found the performance decreased significantly with a decrease in the number of weights, the fading memory of the reservoir was likely to have been only a few input steps.

Beside the number of weights, other factors affected the performance of this task. For example, the amplitude of the input signal $\Delta H$, Fig 6. An optimal value of $\Delta H$ was found at ${5000}\,\text {A/m}$. For typical values of $u_{nk} = [0,1]$ this gave a total *H* that placed the system in a dynamical regime just after the region of chaotic dynamics. We found that on average (over all the utterances) the range of *H* was approximately 1400 A/m to 3100 A/m using this value of $\Delta H$. We noted an overall reduction of the recognition rate by increasing $\Delta H$. This can be correlated to the oscillator response shown in the bifurcation diagram becoming flatter as *H* increased, and thus the relative non-linearity decreasing with respect to $H_0$.

We also explored the effect of $H_0$ on classification accuracy. Figure 6b presents the classification accuracy as a function $\Delta H$ for 5 values of $H_0$ using 1024 weights. Decreasing $H_0$ caused a slight increase in the maximum accuracy obtained while the maximum moved to higher values of $\Delta H$. Furthermore, for low values of $H_0$ a substantial reduction in classification accuracy was observed for low values of $\Delta H$, which was due to the DW operating in the chaotic regime of dynamics. The shift in optimal $\Delta H$ was again due to the offset of the input values, i.e when $H_0$ was lower a larger $\Delta H$ resulted in a field range that existed on the edge of the chaotic region of the bifurcation diagram. With a larger $H_0$ the field range was in the non-linear regime, but again the relative flattening of the system response at high fields ultimately reduced the performance.

#### Chaotic transient mapping for non-temporal tasks

Finally, we demonstrated the ability of the chaotic oscillator to map inputs to a higher dimensional representation for non-temporal tasks. This task used a modified form of the RC method as introduced by Jensen et al.^36^ and is given in more detail in the Methods section. In contrast to the previous RC methods where an internal memory of the reservoir was necessary to solve the tasks, the data sets used here were independent samples and so it was possible to reset the DW position while transforming the input. In this way the transient dynamics were exploited to transform the input into a set of transformed data points that varied non-linearly with the input. By transforming the input into a higher-dimensional space in such a non-linear way it was then solvable with a linear classifier. Instead of changing the field magnitude every $\theta$ it was constant for each input component and the DW dynamics were sampled *k* times per cycle for $N_c$ cycles to build up the transformed vector. After the resulting transformation a linear perceptron was fitted using ridge regression. Here, fivefold cross validation was used to select the L2 regularization parameter ($\lambda$) while performance was measured on separate test parts of the data sets.

**Figure 7:**
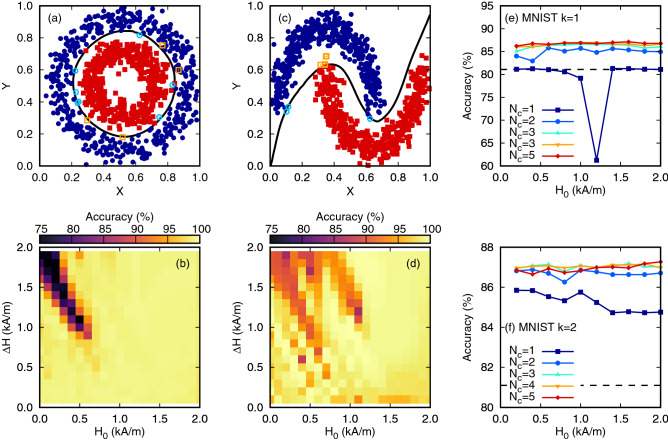
Classification of non-temporal data sets: (**a**,**b**) concentric circles, (**c**,**d**) interlocking half moons and (**e**,**f**) handwritten digits from the MNIST data set. In (**a**) and (**c**) the input is normalised to the range [0,1] and $H={600}\,\text {A/m}$, $\Delta H = {400}\,\text {A/m}$, $N_\text {cyc} = 2$ and $k=2$. The filled points show correct classifications while open orange squares and light blue circles are incorrect. The dashed line shows the computed decision boundary using the DW oscillator. (**b**) and (**d**) Show the accuracy computed on the test set over the range of base field and amplitude values for the circles and half moons data sets respectively. (**e**) and (**f**) Show the classification performance on the MNIST data set using 20 principal components using $k=1$ or 2 samples per cycle respectively. In both $\Delta H = {400}\,\text {A/m}$ is used. The dashed line shows the performance of a linear perceptron of 81.1% on the reduced input data.

We tested the performance on two toy 2D datasets (concentric circles and interlocking half moons) and a more complex case of hand written digits. The first toy data set had each class clustered around a separate ring with additional noise to add complexity. This toy dataset was generated from the Scikit-Learn Python library with a noise value of 0.1 and a scale factor of circle diameters of 0.5. Figure 7a shows an example of this dataset and the predicted classification when using the oscillator to map the input values with $H={600}\,\text {A/m}$, $\Delta H = {400}\,\text {A/m}$, $N_\text {c} = 2$ and $k=2$. The two classes are shown as blue circles and gold squares respectively and points that were correctly classified are shown as solid points while incorrect classifications are shown as open points. The decision boundary predicted by our model is shown as a solid line and with this non-linear mapping the DW oscillator managed to achieve close to 100% accuracy. Figure 7b shows the accuracy surface over the range of $H_0$ and $\Delta H$ parameters. There was a consistent high performance even beyond the chaotic region, where the high degree of non-linearity was expected to aid separation of the two classes. We attributed the performance for high fields to the non-linear variation of the oscillation amplitude which occurred within the first few transient cycles. The region of poor performance appeared to correspond to cases where the input was mapped over a wide range of *H* containing both chaotic and non-linear behaviour.

The second toy data set was composed of two clusters that formed semi-circle shapes that inter-locked. Again this was generated from the Scikit-Learn library with a noise factor of 0.5. The dataset is displayed in Fig. 7c with the same scheme as the first dataset. Again the mapping provided by the oscillator managed to classify the data points with close to 100% accuracy. For both datasets without the oscillator the readout layer would only be able to create a linear decision boundary which would fail to classify many of the datapoints. Figure 7d shows the performance over a range of field parameters for this second dataset. Similar to the first dataset, the performance was lower for low $H_0$ and high $\Delta H$ but beyond $H_0 = {1}\,\text {kA/m}$ consistent high accuracy was achieved.

We also applied this method to the MNIST dataset to test the DW oscillator’s performance on a more realistic task. The MNIST dataset contains 60,000 images of hand written digits (0–9) sampled on to a 28 × 28 grid. For testing purposes, we employed principal component analysis (PCA) to reduce the input dimension to 20 principal components before applying the DW oscillator. In this case we included up to 5 cycles of the transients for each dimension and fixed the input field range to 400 A/m. Figure 7 shows the relative performance over a range of base field values and number of cycles for (e) $k=1$ or (f) $k=2$ samples per cycle respectively. The performance of the linear perceptron without the oscillator for 20 principal components was 81.1%, shown as a dashed line in both panels. The oscillator showed an improvement over the perceptron of approx. 5.8%, increasing as more cycles and samples per cycle were used. As with the toy datasets the performance was relatively unchanged over the range of field values considered, except in the case of $k=1, N_c=1$ where there was decrease in performance at $H_0 = {1.2}\,\text {kA/m}$. In this case the oscillator was mapping the input to only 1 virtual neuron and in this range of field the transformation of the input was approximately quadratic (i.e $x \approx (u-0.4)^2$). Whilst this non-linearity should be beneficial it appeared to reduce the classification accuracy since it allowed for similar outputs for different inputs (e.g $x(u=0.2) \approx x(u=0.6)$). Together these results demonstrated the power of using the DW oscillator as a reservoir to map non-temporal tasks to a higher dimensional, non-linear representation.

## Conclusions and further work

In this paper we have demonstrated that the dynamics of individual, geometrically-confined DWs are suitable for use as hardware-based reservoirs in neuromorphic computing applications. We have used both 1D collective coordinates models and micromagnetic simulations to show that such reservoirs would exhibit good performance in both temporal and non-temporal tasks including spoken and written digit recognition. We have also investigated the fundamental dynamics of DW oscillators, and explored how the non-linearity of these relate to its performance in the classification tasks. Together our results create a compelling case that individual, nanoscale DWs have dynamics suitable for creating neuromorphic computing devices with critical dimensions $<{1}\,\upmu \text {m}$.

Further work will be required to better understand the feasibility of creating real devices based on DW oscillators. For example, here we have used an oscillating magnetic field to drive DW dynamics, but in a real device either spin torques or spin-orbit torques would need to be used to improve energy efficiency. This would also likely require a move to materials with out-of-plane anisotropy where these effects are strongest^37,38^. Device readout will also require further development. In our simulations we have used the position of the DW as an output parameter. In a real device this could be achieved by integrating the oscillators into spin-valve stacks, where the device resistance would be directly proportional to the position of a DW. However, accurate measurements of this in a ~ GHz frequency device may prove challenging. Furthermore, the effects of both thermal perturbations and lithographic defects, both features of any real device, on performance will need to be explored. In particular, it is likely that thermally-induced randomness will degrade classification performance at all field amplitudes, although training weights so as to maximise the margins between the decision boundaries and reservoir outputs might reduce this error as the weights learn to generalise the data better. Additionally, at low applied fields stochastic resonance may mask the chaotic behaviour observed in the deterministic model^39^ (although we note that the chaotic behaviour is not essential for successful classification in any of the tasks we’ve explored here). Establishing how DW oscillators can be tuned to mitigate these effects will be important to future device proposals.

DW oscillators have several features that may make them powerful as reservoirs. For example, their dynamics are fundamentally controlled by the shape of their confining potentials; these could be easily modified by altering device geometry or material properties in order to tune dynamics for high performance in a given application. Furthermore, it may be possible to create systems where magnetostatic interactions are used to couple closely-spaced DW oscillators in parallel nanowires. This could allow the creation of multi-input, multi-output reservoirs with far greater computational power than the simple, single neuron reservoirs we have explored here.

## Methods

### Domain wall oscillator model

In the collective coordinates model, it was assumed that the DW structure did not vary substantially during motion (i.e. the DW type did not change) and so the DW could be described by a pair of collective coordinates: the position of the DW centre along the nanostrip (*X*) and the tilt angle of the DW centre ($\psi$). The equations of motion of these collective coordinates^30,40,41^ were:

1 $$\begin{aligned} \frac{(1+\alpha ^2)}{\gamma \mu _0} {\dot{\psi }}&= H_\text {p}(X) - \frac{\alpha }{2} H_k \sin (2\psi ) + H \sin (2 \pi f t), \end{aligned}$$

2 $$\begin{aligned} \frac{{\dot{X}}}{\Delta }&=\frac{\gamma \mu _0 H_k}{2}\ \sin (2\psi ) - \alpha {\dot{\psi }} , \end{aligned}$$

where $\alpha$ was the material damping constant, $\gamma$ was the gyromagnetic factor, $\mu _0$ wsa the magnetic permeability of free space, *S* was the nanowire transverse cross section, $M_s$ was the saturation magnetization, $\Delta$ was domain wall width parameter and $H_k$ was the nanowires’ shape anisotropy field. The final term of Eq. (2) represented a sinusoidally varying externally applied magnetic field with frequency *f* and magnitude *H*. $H_\text {p}$ was the effective field arising from the interaction of the DW with the anti-notches and was defined as

3 $$\begin{aligned} H_\text {p}(X) = -\frac{1}{2\mu _0 M_s S} \frac{dE(X)}{d X}, \end{aligned}$$

where *E*(*X*) represented the position-dependent energy of the DWs, which contained the contributions from both the exchange and demagnetizing energy. *E*(*X*) was previously calculated using micromagnetic simulations by Martinez et al.^31^. For symmetric anti-notches, *E*(*X*) was approximated by a double-well potential of the form:

4 $$\begin{aligned} E(X) = a X^2 + b X^4. \end{aligned}$$

The coefficients *a* and *b* were fitted to best represent the micromagntic potential calculated by reference^30^, which resulted in $a=-1.28\times 10^{-6}\,\text {Jm}^{-2}$ and $b=1.63\times 10^{8}\,\text {Jm}^{-4}$.

The domain wall width, $\Delta$, was calculated analytically using

5 $$\begin{aligned} \Delta =\pi \sqrt{\frac{2A}{\mu _0 M_s^2 \sin ^2 \psi + \mu _0 M_s H_k}}, \end{aligned}$$

where *A* is the exchange stiffness.

The nanowires’ shape anisotropy field $H_k$ was introduced analytically using^42^:

6 $$\begin{aligned} H_k=M_s(N_z-N_y), \end{aligned}$$

where $N_z$, $N_y$ were the demagnetization factors on *z* and *y*-axis^43^.

We used standard parameters for the material properties of nickel: saturation magnetization $M_s=470$ kA/m, exchange stiffness $A=1.05\times 10^{-11}$ J/m and damping parameter $\alpha = 0.02$. It was assumed that nanowire was polycrystalline and exhibited no net magnetocrystalline anisotropy.

The above system of equations was integrated numerically using the fourth-order Runge-Kutta technique in order to find the space-time evolution of the DW. We used an optimized integration step of $10^{-13}$ s for which the dynamics converged for a wide range of damping values. To verify the accuracy of the collective coordinates model we performed simulations using the MUMAX3 GPU-accelerated software package^44^. The nanowire was descretized into ${2.5}\,\text {nm} \times {2.5}\,\text {nm} \times {2.5}\,\text {nm}$ cells. The DWs were initially relaxed in the centre of the nanowire, prior to magnetic fields being applied.

### Reservoir computing

In reservoir computing, the expensive training of a fully connected recurrent neural network is avoided by instead using a fixed reservoir to transform the inputs such that the transformed representation can be fitted by a linear model (i.e perceptron). For systems with only a single input and output a method for RC was proposed by Appeltant et al.^32^ based on time multiplexing of the input and output signals. This method has been used widely for RC with single dynamical objects and in particular by Torrejon et al.^7^ to perform reservoir computing with a single magnetic tunnel junction. In this method rather than having multiple physical neurons to input and readout from, the system state at different time intervals was used as ‘virtual’ neurons i.e. input/output was multiplexed in time. In this way the virtual neurons were connected sequentially in time and each input dimension was randomly combined to drive the reservoir dynamics at each virtual neuron. In full the method consisted of the following steps:

- The signal was pre-processed using any relevant methods (e.g spectrogam) and split into $N_s$ samples, such that $u_k(n \delta t) = u_{nk}$ was the value of the *k*-th input dimension for sample *n*. This input signal had a dimension of $N_\text {in}$ and so the full input matrix had a size of $N_s$ by $N_\text {in}$. For training, these inputs corresponded to a desired output value, $Y_{ni}$, which again was formed into a matrix of shape $N_s$ by $N_\text {out}$, where $N_\text {out}$ was the number of possible outputs (i.e classes).
- Each input sample vector was broadcast to the virtual neurons by applying a random binary input mask. This took the form of a $N_v$ by $N_\text {in}$ matrix where the elements were $m_{jk} = \{-1, 1\}$. The processed signal was then scaled to give the input magnetic driving field for the reservoir as
  7 $$\begin{aligned} H_{nj} = H_0 + \Delta H \sum _{k=1}^{N_\text {in}} m_{jk} u_{nk}, \end{aligned}$$
  where $H_0$ was the base amplitude of the driving field and $\Delta H$ was the amplitude of the stimulus. The index *j* is that of the virtual neuron and *k* is summed over the input dimension.
- The input magnetic fields were then serialized into a 1 dimensional time sequence which was applied to the reservoir in turn and held for the virtual neuron duration, $\theta$.
- At the end of each virtual neuron time segment the reservoir state, i.e DW position *X* or RMS position, was recorded and formed into a matrix of output values which had a shape of $N_s$ by $N_v$, such that the transformed value for sample index *n* and virtual neuron *j* was
  8 $$\begin{aligned} X_{nj} = X(n\tau + (j+1)\theta ). \end{aligned}$$
- Finally using this readout, the predicted output for sample *n* and output dimension *i* was given by
  9 $$\begin{aligned} {\tilde{Y}}_{ni} = \sum _{j=1}^{N_v} W_{ij} X_{nj} + b_i, \end{aligned}$$
  where $W_{ij}$ were the elements of the linear output weight matrix and $b_i$ the bias for output dimension. In practice, the bias was included as part of the weight matrix as $W_{i0} = b_i$ with the *X* matrix augmented such that $X_{n0} = 1$ for all *n*.

The aim of the supervised learning process was to train the output weights to minimize the difference between the predicted output and the desired outputs. This was achieved by minimizing an appropriate error function, which for simplicity the mean squared error (MSE) with a L2 weight penalty term was used^5^. The MSE was defined as

10 $$\begin{aligned} MSE = \sum _n \sum _i (Y_{ni} - {\tilde{Y}}_{ni})^2 - \lambda \sum _{ij} W_{ij}^2, \end{aligned}$$

where $\lambda$ was a constant that controls the L2 penalty term. This was a hyper-parameter that was chosen through a grid search approach to find the value with the lowest MSE on the test part of data set. The penalty term limited the magnitude of the weights and stopped over-fitting the data. By using this error function the weights could be solved by using the ridge (or Tikhonov) regression method, such that the weights were computed using

11 $$\begin{aligned} {\mathbf {W}} = {\mathbf {Y}}^T {\mathbf {X}} \left( {\mathbf {X}}^T {\mathbf {X}}+ \lambda {\mathbf {I}} \right) ^{-1}, \end{aligned}$$

where ${\mathbf {I}}$ was the identity matrix and the matrix inverse was approximated using the pseudo-inverse. For further details on the reservoir computing approach, including the regression method for learning the weights, we direct the readers to Ref.^5^.

The amplitude of the driving field at each virtual neuron time segment was chosen to be the input while the frequency was kept fixed. Using this method for reservoir computing, there are several hyper-parameters which set the operating regime; $H_0$, $\Delta H$, $\theta$ and $N_v$. $H_0$ and $\Delta H$ set the field range for which the oscillator was acting in and thus how chaotic or non-linear the output was. $\theta$ was the duration of each virtual neuron and thus was related to the rate of input to the system. Since the virtual neurons were connected sequentially in time, to make use of any internal memory of the system $\theta$ had to be shorter than the fading memory time of the system. As we show in the results section, optimising these parameters led to a significant improvement in the performance.

### Non-temporal classification

An alternative method to transform the input values for non-temporal tasks is to use the transient dynamics from a fixed initial state, as described by Jensen et al.^36^ Since the device started from a fixed point it had no internal memory but the non-linear dynamics could transform the input such that it was separable by a hyper-plane.

As in the previous methods, each input sample had a dimension of $N_\text {in}$ and the *n*-th sample was represented by the vector ${\mathbf {u}}_n = \left[ u_{n1} \dots , u_{ni}, \dots , u_{nN_\text {in}} \right]$. The aim of this method was to transform each element of the input sample separately and concatenate the resulting output vectors to give a full transformed output sample, ${\mathbf {X}}_n = \left[ {\mathbf {X}}_{n0}, \dots , {\mathbf {X}}_{ni}, \dots , {\mathbf {X}}_{n N_\text {in}}, \right]$. Using this fully transformed vector a linear output perceptron was trained to classify the samples using the same ridge regression method given in Eq. (11).

The transformation process was as follows. First, the input data was rescaled so that each component was in the range [0, 1]. For each component of the input sample the DW position and tilt angle were reset to zero before being evolved for $N_c$ cycles. The field magnitude was constant over all the cycles with a value of $H_{ni} = H_0 + \Delta H u_{ni}$ for the *i*-th component of the *n*-th input sample with $H_0$ and $\Delta H$ again taken as scaling hyper-parameters. The DW position was sampled *k* times per cycle (i.e the neuron duration was $\theta = 1/ kf$) over this evolution to give $N_v = k N_c$ virtual neurons for each component of the input. The sampled DW position for each component of the input was joined to give a final output vector of size $N_\text {in} k N_c$ to which a linear perceptron was fitted.

## Data Availability

Our 1D model code is freely available on github repository by accessing following link: https://github.com/maxxwave/DWPC.
